# Optimization of technique for insertion of implants at the supra-acetabular corridor in pelvis and acetabular surgery

**DOI:** 10.1007/s00590-017-2007-8

**Published:** 2017-06-28

**Authors:** Theodoros H. Tosounidis, Cyril Mauffrey, Peter V. Giannoudis

**Affiliations:** 1 Academic Department of Trauma & Orthopaedic Surgery, University of Leeds, Clarendon Wing, Floor A, Great George Street, Leeds General Infirmary, Leeds, LS1 3EX UK; 2 NIHR Leeds Biomedical Research Unit, Chapel Allerton Hospital, West Yorkshire, Leeds, LS7 4SA UK; 3 Denver Health Medical Center, Denver, CO USA

**Keywords:** Pelvis, External fixator, Supra-acetabular external fixator, Anterior inferior iliac spine

## Abstract

The technique for application of implants at the sciatic buttress has been well described in the pelvic and acetabular fracture reconstruction literature. We described a new use of the inlet–obturator oblique view for the identification of the anterior inferior iliac spine, which is the entry point of implants, and we provide a detailed fluoroscopic and radiographic description of this view. A small series of 15 patients who underwent an application of an anterior inferior pelvic external (supra-acetabular) fixator via this technique is presented. We consider the use of the obturator oblique for the identification of the entry point unnecessary, and we advocate for the use of only the inlet–obturator oblique and iliac oblique views when implants are applied to the sciatic buttress.

## Introduction and background

The sciatic buttress is commonly used as an osseous fixation pathway in pelvic and acetabular fracture reconstruction [1]. It is a long, tubular-shaped structure that can accommodate implants in internal and external fixation reconstruction procedures, extending between the anterior inferior iliac spine (AIIS) and the posterior inferior iliac spine (PIIS) [2]. The technique for the application of these implants has been extensively described in the literature [3–8]. The current practice of insertion of implants in tubular bones (humerus, ulna, radius, femur, tibia, fifth metatarsal) mandates that two orthogonal fluoroscopic views of the entry point be obtained. Nevertheless, according to the current technique of the supra-acetabular implant insertion, the entry point (AIIS) is visualized only in one view, which is the iliac oblique (IO). The other view utilized during the identification of the entry point is the obturator oblique (OO) view, which actually presents a tangential visualization of the supra-acetabular corridor and not of the AIIS [4].

In the herein study, we present an optimization of the technique of implant insertion using the inlet–obturator oblique (IOO) view for the accurate identification of the AIIS and we describe the radiological landmarks of this view. We consider the use of the obturator outlet view as unnecessary, and we suggest that the entire procedure can be safely and effectively performed by the use of the IOO and IO views.

## Surgical technique and clinical experience

**Fig. 1:**
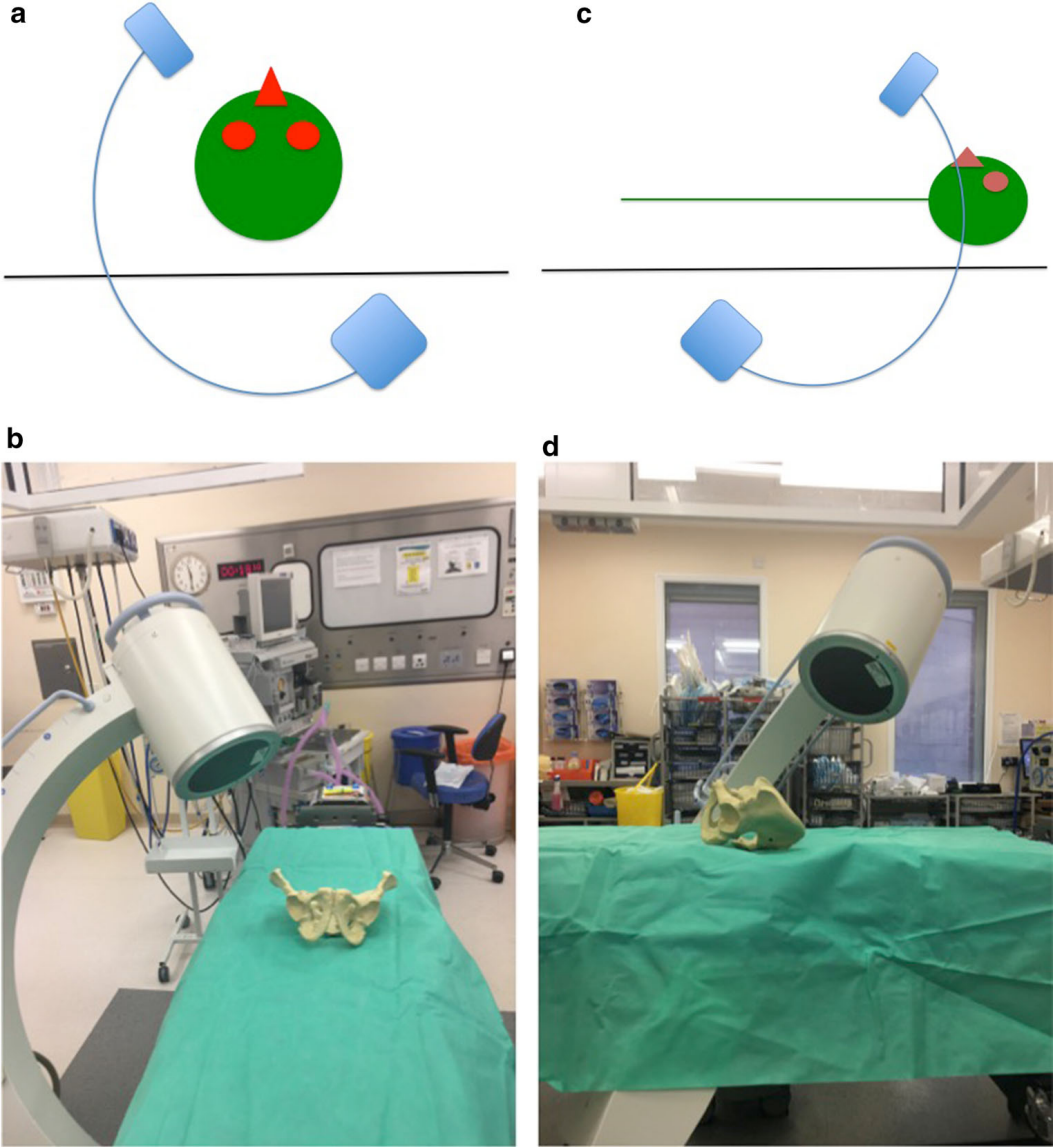
Schematics and photographs demonstrating the position of the image intensifier **a**, **b** in the obturator oblique and **c**, **d** in the inlet elements of the view with the patient supine

**Fig. 2:**
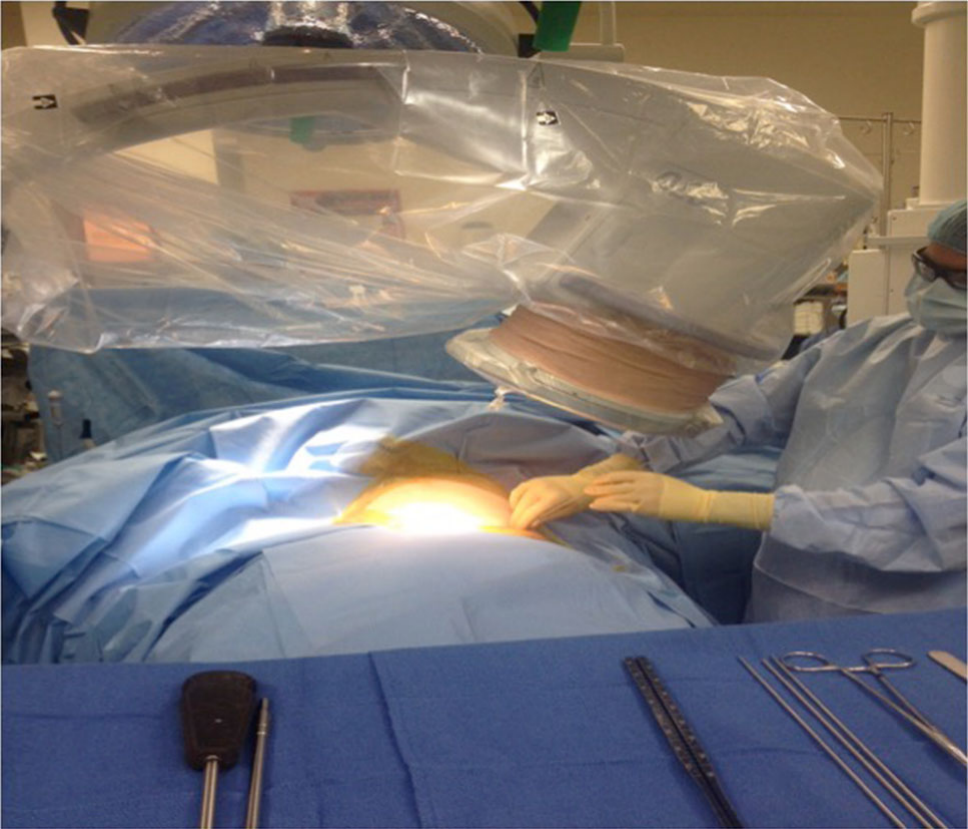
Clinical photograph demonstrating the position of the fluoroscopy unit in relation to the position of the surgeon during the utilization of the OO view. Note that the very limited space for maneuvring making the application of the half pin challenging

**Fig. 3:**
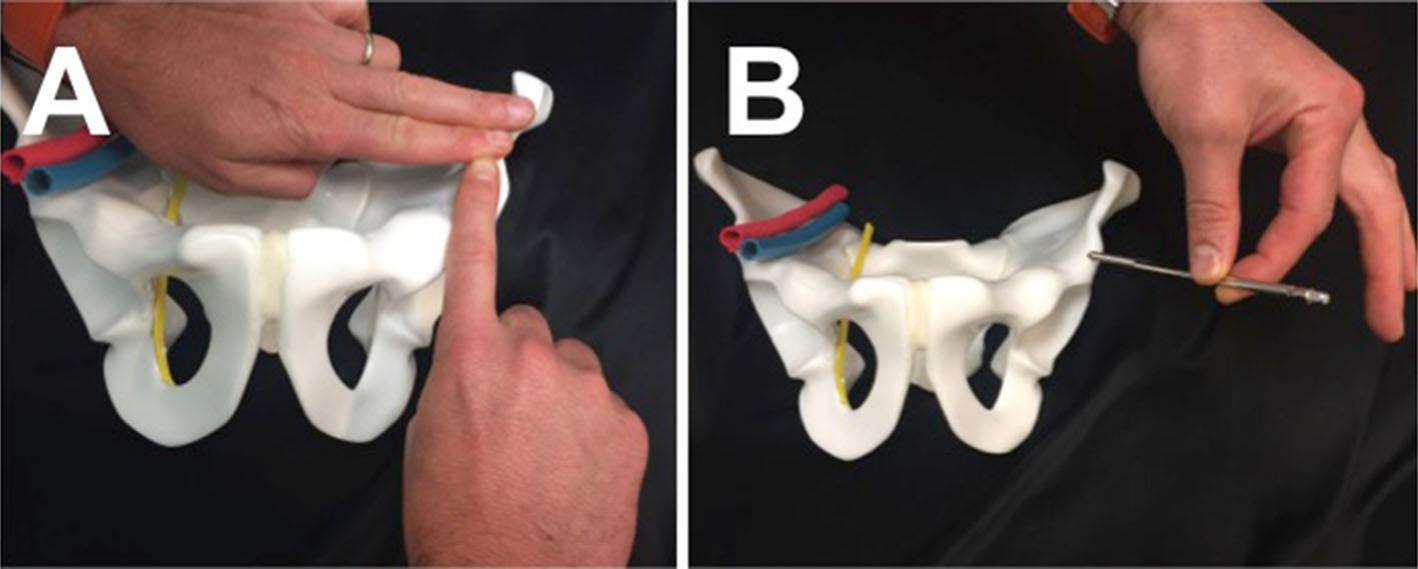
Identification of the entry point two fingerbreadths distal and one medial to the ASIS

**Fig. 4:**
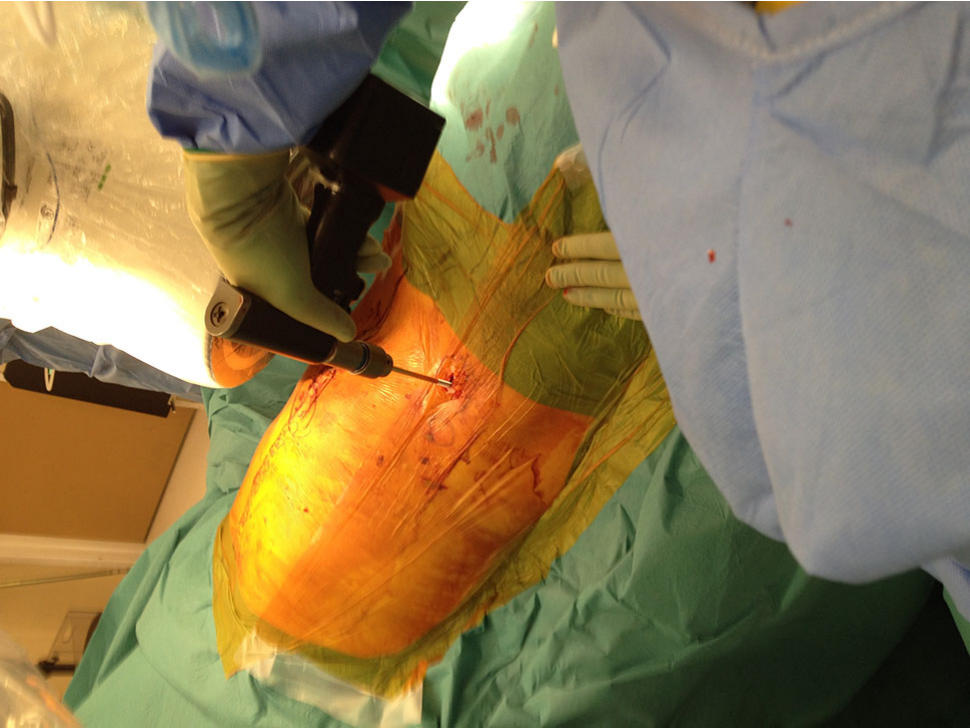
Clinical photograph demonstrating the trajectory of the half pin at the sagittal and axial planes

**Fig. 5:**
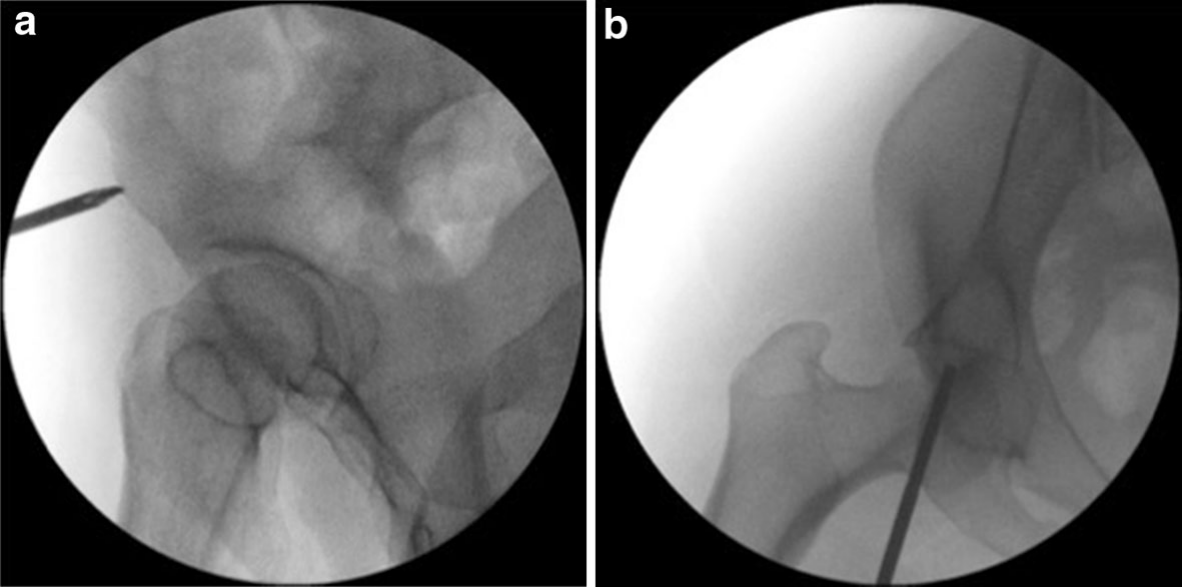
Insertion of a supra-acetabula external fixator pin using the technique without utilizing the OO view: The entry point of the AIIS tip is identified using **a** the IO and **b** the IOO views

The patient is positioned supine on a radiolucent table. The image intensifier is brought from the ipsilateral to the injury side (Fig. 1). Standard preparation and draping of the surgical field then follows. The umbilicus and the midline are marked. The osseous landmark used for insertion of implants to the AIIS is the anterior superior iliac spine, which is easily palpable. To identify the AIIS, an outlet obturator oblique is usually required. However, this view may be challenging to obtain with the external fixator pin and the surgeon’s hand in the way (Fig. 2). We therefore mark the ASIS and a 1-cm longitudinal skin incision is made two fingerbreadths distal and one medial to the tip of the ASIS (Fig. 3a, b). Tonsils are utilized to dissect bluntly, the interval between the sartorius and the tensor fascia lata muscles is palpated with a blunt instrument, and deep dissection takes place. The ideal entry point is located just above the rectus femoris insertion at the AIIS [8–12]. A 200 mm × 5 mm external fixator pin is then positioned using tactile feedback for the identification of the medial and lateral edges of the AIIS. A mallet is utilized to tap the pin in place just a few mm deep, aiming approximately 40 degrees cephalad and 40 degrees medial (Fig. 4) depending upon the pre-existing pelvic deformity. In our technique, the next step is the identification of the entry point using an iliac oblique view (Fig. 5a). In this view, the half pin should be placed at least 1.5–2 cm above the hip joint in order to avoid penetration of the hip capsule. The AIIS is visualized as a curve with the convex aspect facing anteriorly. The half pin is placed at the tip of the convexity and the trajectory is checked in relation to the sciatic notch.

**Fig. 6:**
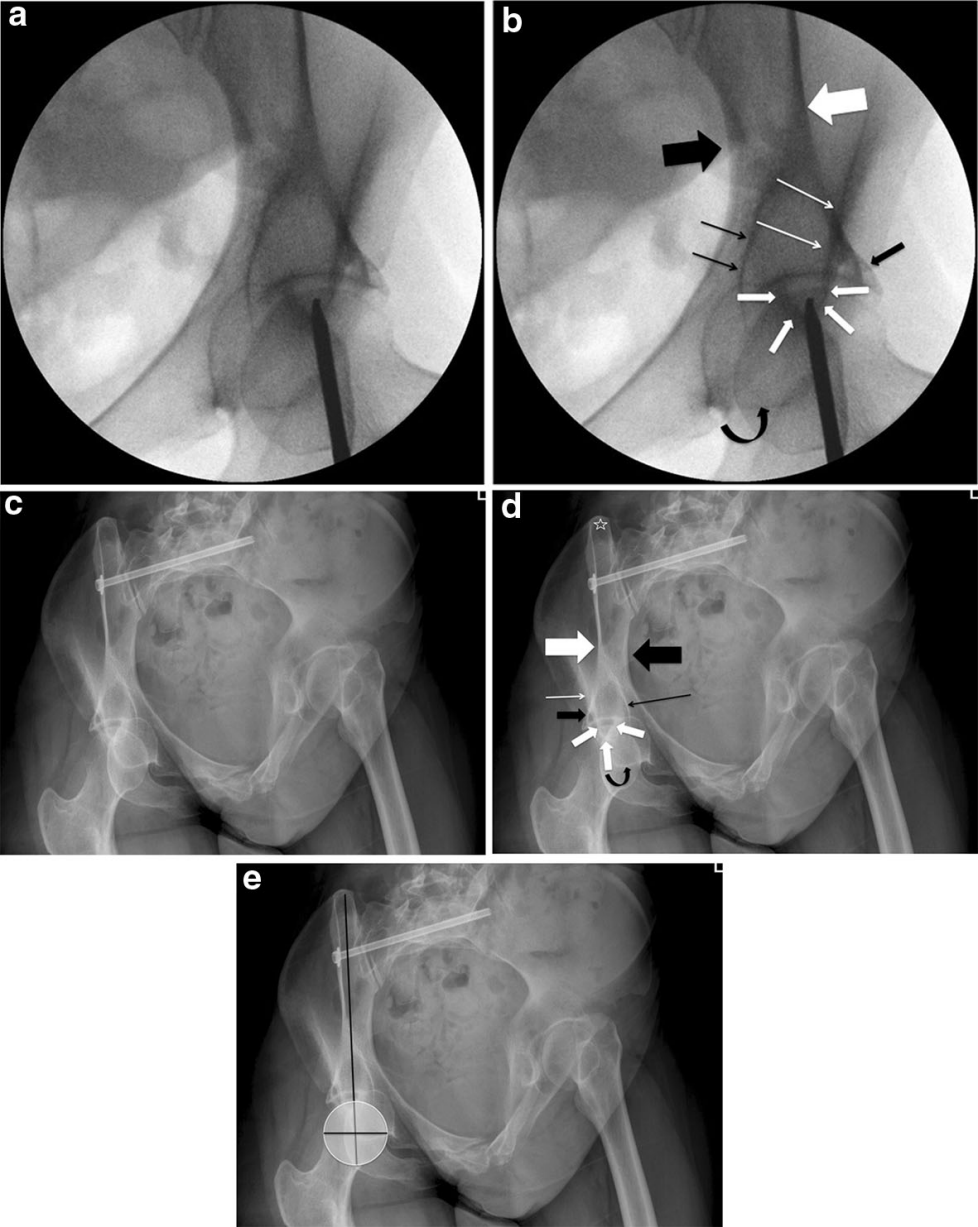
**a**, **b** Fluoroscopic and **c**, **d** radiographic views of the inlet and obturator oblique views. The following anatomical structures can be identified: anterior inferior iliac spine (intermediate *thickness white arrows*). Inner table of sciatic buttress (*thick black arrow*). Outer table of sciatic buttress (*thick white arrow*). Outer aspect of supra-acetabular area (*thin white arrow*). Inner aspect of the posterior column (*black thin arrow*). Ischium (curved *black arrow*). Posterior inferior iliac spine (*white star*). **e** In the “ideal IOO view,” the straight line connecting the AIIS to PIIS should bisect the femoral head. The AIIS is projected overlapping with the proximal middle part of the femoral head

The next step in our technique is to obtain an inlet–obturator oblique view (Fig. 5b). With the patient in the supine position, the image intensifier source should be moved toward the patient’s head and toward the affected acetabulum/hemipelvis. This view allows for tangential visualization of the AIIS and offers safe definition of its inner and outer margins. Additionally, it offers visualization of its most anterior (i.e., prominent) point. In this view, the AIIS is visualized as a “thumb-like” structure with its concavity directed posteriorly and is located in the middle part of proximal half of the femoral head. The outer aspect of the AIIS is better defined compared to the inner one, as it extends more posterior compared to the inner. This line represents the supra-acetabular cortex from AIIS to posterior edge of the gluteal pillar. The femoral head, the AIIS and the posterior column are overlapping. The inner part of the posterior column is shown as an elliptical line with the concave facing outwards, medially to the AIIS and laterally to the pelvic brim which runs from posterior to anterior direction. The ischium is also shown overlapping with the femoral head, medially and anterior to the AIIS. The various anatomical structures that can be identified in IOO view are the inner and outer tables of the sciatic buttress, the retroacetabular surface, the acetabular dome and the obturator foramen (Fig. 6a–d).

**Fig. 7:**
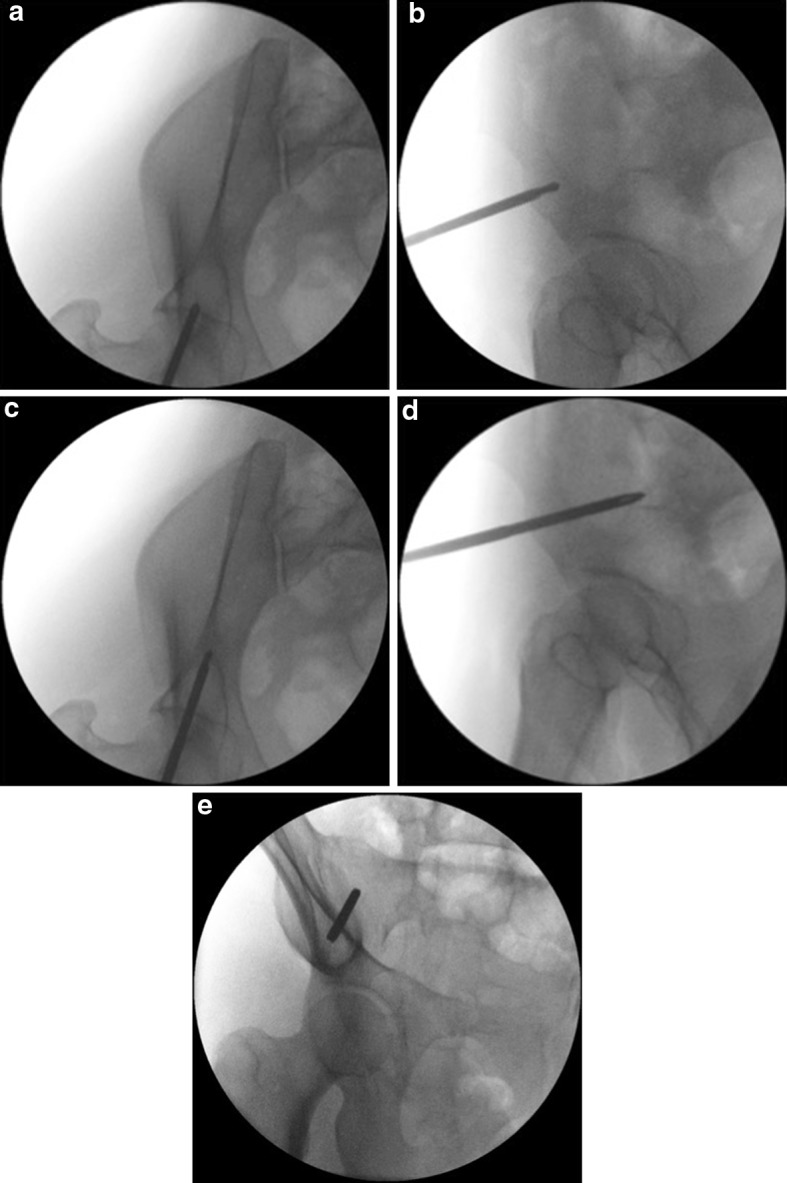
**a**–**d** Advancement of the pin is taking place using the same views IOO and II views. **e** OO view performed after the pin placement

The view must be adjusted according to the pre-existing pelvic deformity. In the intact or minimally displaced pelvis, the usual range of inclination is 30°–45° in the inlet view and 30°–45° in the obturator view. The ideal IOO view shows the AIIS in its widest axial dimension, i.e., the distance between the inner and outer tables should be the maximum possible. This can be achieved by customizing the tilt of the C-arm. In the ideal IOO view, both the AIIS and the posterior inferior iliac spine should be visible. A straight line connecting these structures should lie within the inner and outer boundaries of the sciatic buttress and also vertically bisect the femoral head. As previously mentioned, the AIIS should be visualized overlapping the femoral head and should be ideally seen on the middle upper section of it (Fig. 6e). Over- and/or under-inclination as well as over- and/or under-rotation can be avoided by ensuring that the above prerequisites about the “ideal IOO view” are met. After the correct identification of the entry point, the half pin is advanced using the IOO and the II views (Fig. 7a–e).

**Fig. 8:**
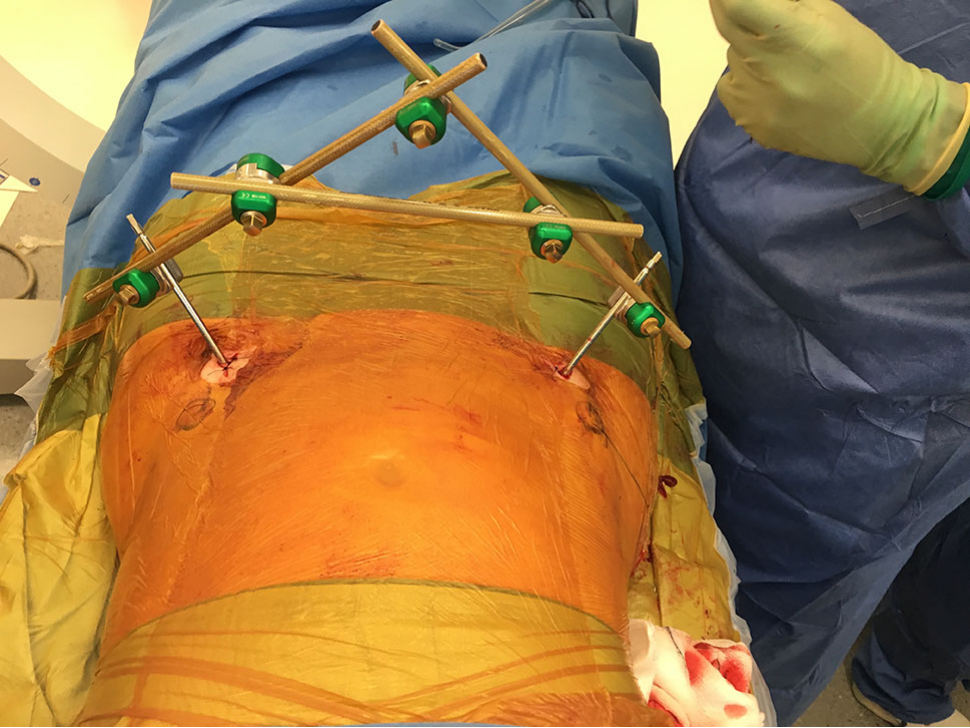
The final construct of an anterior inferior external fixator. The entry point two fingerbreadths distal and one medial to the ASIS is obvious

We have used this technique in a series of 15 consecutive patients who suffered a pelvic insufficiency fracture (lateral compression type 1) [13] that was treated with an anterior distraction frame and percutaneous sacroiliac screws (Fig. 8). All the patients had suffered a complete sacral fracture and during the intra-operative examination under anesthesia they demonstrated instability (more than 2 cm movement of the ipsilateral pubic ramus fracture) [14]. In these patients, the pelvic EXFIX was applied for a period of 4 weeks before it was uneventfully removed at the orthopedic outpatient clinics. An obturator outlet view was obtained at the end of the half pin application to confirm its “intra-osseous” placement. No misplacement was observed. One patient developed a transient meralgia paresthetica due to irritation of the lateral femoral cutaneous nerve that was resolved within 3 weeks after the external fixator removal. The same patient also suffered a superficial pin site infection that was treated with a short course of oral antibiotics and pin site care.

## Discussion

The sciatic buttress is an osseous fixation corridor that is commonly used in pelvic and acetabular surgery for the application of half pins of an anterior inferior (supra-acetabular) external fixator [3–6, 15], of the pedicle screws of anterior pelvic internal fixator (INFIX) [8, 16] and in internal fixation procedures in pelvic and acetabular fracture surgery as part of the anterior column fixation [1, 17, 18]. It is also used in spinal fixation for the application of screws through the posterior iliac spine [19]. The entry point for insertion of implants to this osseous corridor is the AIIS. The relevant anatomy of the area as well as the relation of the inserted implants to the vital anatomical structures has been described in anatomical and radiology studies [2, 9, 10, 16, 20–22]. The above-mentioned publications described the details of the technique for safe insertion of implants to the sciatic buttress. According to this technique, the combination of obturator and outlet view is used initially to define the entry point. In this view, the so-called "tear-drop" is identified by adjustment of the angle of the C-arm in the two planes until directly parallel to the osseous corridor of the sciatic buttress. After the correct identification of the starting point, the procedure then continues with visualization of the bone in two orthogonal planes. The fluoroscopic views used for this are the iliac oblique and the inlet obturator.

The sciatic buttress is a tubular structure, and the fluoroscopic insertion of implants should follow the rules of implant insertion in other tubular structures such as intra-medullary nailing of the femur and tibia [23, 24]. In these procedures, the starting point is not identified by trying to obtain a perfect tangential fluoroscopic view of it. Instead, it is identified by using two orthogonal views. For example, the starting point of a trochanteric entry nail is identified using an anteroposterior and a lateral view of the proximal femur and not by trying to obtain a tangential view of it. The only exception of this technique is encountered in the application of spinal pedicle screws. In this situation, an orthogonal to the lateral view is impossible and a tangential to the pedicle projection is obtained to identify the entry point [25].

In an effort to extrapolate the above practice in the application in the sciatic buttress, we describe the use of the IOO view, which makes the fluoroscopic visualization of the AIIS easy. We advocate for the use of this view, and we think that the obturator outlet view is unnecessary. We support that the IOO view should be used to identify the correct starting point for the following reasons:

1. It is easy to obtain and familiar to all orthopedic surgeons who are treating pelvic and acetabular fractures. This view has been used mainly to define the safe corridor for the application of implants that are directed from the anterior inferior iliac spine to the posterior inferior iliac spine or vise versa [4, 19]. This view is also used to define the outer cortex of the iliac bone in the application of iliosacral screws and prevent the penetration of its outer cortex or leaving the screws proud [26]. It has also recently been described for the safe application of implants during the fixation of posterior wall fractures [27].
2. It offers clear tangential visualization of the AIIS and thus reassurance that the implants are away from the main anatomical structures at risk (i.e., the femoral nerve).
3. It obviates the use of the obturator oblique view and makes the entire procedure faster. Additionally, the fluoroscopy C-arm is always away form the surgeon, thus minimizing the risk of de-sterilization during the procedure. Although not tested in this study, it is reasonable to assume that the above benefits might be useful in situations when time is of essence, e.g., when the anterior inferior EXFIX is used for stabilization of a hemodynamically unstable patient. Nevertheless, we appreciate that we have not documented the surgical time and the radiation required for insertion of supra-acetabular screws using this technique.
4. Additional to the identification of the starting/entry point, it can be simultaneously used to define the trajectory of the implant. It is our observation that the most technically demanding and time-consuming step during the application of an anterior inferior EXFIX is to maintain the correct trajectory of the drill bit during the transition of one fluoroscopic view to another at the beginning of the operation, i.e., in defining the correct entry point. This is because the AIIS is a small convex structure offering small purchase surface area for safe anchoring of the implants. By using the IOO view instead of the OO view, the correct trajectory is defined from the very beginning. Furthermore, avoiding tilting the top of the C-arm caudally facilitates the maintenance of the correct starting point once this is obtained. The AIIS is also visible in the classic inlet view of the pelvis. Nevertheless, with this view it is not possible to delineate the trajectory of the implant and the IOO is again necessary.
5. No special instructions/training for the radiographers are necessary, since this view is already being used in the same procedure.

We appreciate that not having assessed the safety of the use of the “IOO” view in cadavers and/or sawbones and/or postoperative CT scans is one of the limitations of the study, which primarily aimed to present the optimization of a known technique and the relevant technical details pertaining to it. However, our clinical experience and nowadays the routine application of this view provides with the confidence that the technique has a short learning curve, is safe and reproducible.

## Conclusion

The IOO fluoroscopic view provides tangential imaging of the AIIS. It can be used along with the IO view to identify the AIIS in two planes. Implants to the supra-acetabular sciatic buttress corridor can be inserted using only the IOO and IO views. The use of the OO view for fluoroscopic identification of the AIIS  is unnecessary. We consider the technique used in this case series useful in the application of both internal (INFIX, internal fixation in acetabular fractures) and external fixation (supra-acetabular EXFIX) implants.
